# Diabetes-induced redistribution of mast cells to the adventitia in ascending aortic aneurysms

**DOI:** 10.17305/bb.2025.12772

**Published:** 2025-07-29

**Authors:** Aleš Pleskovič, Ruda Zorc-Pleskovič, Marjeta Zorc, Aleksandra Milutinović

**Affiliations:** 1 Institute of Histology and Embryology, Faculty of Medicine, University of Ljubljana, Ljubljana, Slovenia; 2 International Center for Cardiovascular Diseases MC Medicor d.d., Izola, Slovenia; 3 Division of Internal Medicine, University Medical Centre Ljubljana, Ljubljana, Slovenia

**Keywords:** Mast cells, MCs, vascularity, type 2 diabetes, DM, arterial hypertension, AH, ascending aortic aneurysm

## Abstract

Mast cells (MCs) are inflammatory cells that reside mainly in the intima of healthy and early-atherosclerotic abdominal aortas but migrate to the adventitia in advanced atherosclerosis and abdominal aortic aneurysms. We compared MC infiltration in the intima, media, and adventitia of ascending aortic aneurysms from patients with diabetes mellitus (DM) or arterial hypertension (AH). Fifty-one patients (36–81 years) undergoing surgical repair were enrolled and allocated to a DM group without AH (*n* ═ 9) or an AH group without DM (*n* ═ 42). Aortic specimens were stained with hematoxylin–eosin and immunohistochemically labeled with anti-CD117 to detect MCs and anti-vWF to visualize blood vessels. Compared with the AH group, the DM group had fewer MCs in the intima and more in the adventitia (Mann–Whitney test, *P* < 0.05). In both groups, intact MCs outnumbered degranulated MCs in the adventitia, whereas no such difference was observed in the intima or media (*P* < 0.05). Medial vascular density did not differ between groups (*P* < 0.05). In the AH group, medial vascularization correlated positively with intact, degranulated, and total MC counts, whereas in the DM group it correlated only with degranulated MCs (Spearman, *P* < 0.05). These findings suggest that DM-associated aneurysms exhibit a distinct MC distribution and vascular response, indicating a pathogenesis that differs from that of AH-associated aneurysms.

## Introduction

Mast cells (MCs) are primarily recognized for their role in mediating immunoglobulin E (IgE)-dependent allergic reactions [1, 2]. However, they also play a significant role in regulating various physiological processes. MCs are crucial for host defense, as they destroy bacteria and parasites. Additionally, they promote vasodilation, angiogenesis, and modulate the activity of diverse immune and structural cells, including macrophages, T and B lymphocytes, eosinophils, endothelial and epithelial cells, and fibroblasts [1].

The cytoplasmic granules of MCs contain numerous bioactive mediators, such as histamine, heparin, proteases, prostanoids, leukotrienes, cytokines, chemokines, and growth factors. Upon activation, these mediators are released to influence tissue and organ function [3]. Beyond their involvement in hypersensitivity reactions, asthma, and anaphylaxis, MCs are implicated in various pathological conditions, including gastrointestinal and cardiovascular diseases [4]. Notably, they contribute to vascular inflammation following hypoxia and ischemia/reperfusion injury, the progression of atherosclerosis, and the development of abdominal aortic aneurysms (AAAs) [2, 4–8].

MCs have been identified in the thoracic and abdominal aortic walls of healthy individuals [9, 10]. In the early stages of atherosclerosis, MCs are predominantly located in the intima, with minimal presence in the adventitia. As the disease progresses, the number of MCs decreases in the intima while increasing in the adventitia. In advanced atherosclerotic lesions and AAAs, MCs are scarce in the intima but become significantly more abundant in the adventitia [9, 11, 12]. In cases of thoracic aortic aneurysm, the quantification of MCs in the adventitia reveals a higher abundance in patients with aneurysms compared to non-aneurysmal controls [10].

Aortic aneurysms most commonly occur in the abdominal segment of the aorta, while thoracic aortic aneurysms are less prevalent. Among the thoracic segments—the aortic root, ascending aorta, aortic arch, and descending aorta—the root and ascending aorta are the most frequently affected sites [13, 14].

The primary risk factors for the development of ascending aortic aneurysms include arterial hypertension (AH), age over 65, and male sex. Epidemiological studies indicate that 4%–8% of men and approximately 1.5% of women over the age of 60 are affected by aortic aneurysms [14–17]. In patients with diabetes mellitus (DM), aortic aneurysms are relatively rare and tend to progress more slowly [18–20].

Our research group has previously examined ascending aortic aneurysms in patients with DM who do not have AH, type 1 DM, or genetic mutations, comparing them to patients with AH but without DM. Our primary focus was on the infiltration of various inflammatory cell types in the intima, media, and adventitia. We found that the intima and media in DM patients exhibited less infiltration. Specifically, DM patients had fewer pro-inflammatory macrophages and B cells in the intima and media compared to AH patients, while the media and adventitia showed a reduced number of plasma cells. No significant differences were noted in the counts of T helper (Th) cells, cytotoxic T (Tc) cells, or anti-inflammatory macrophages [15, 16].

As MCs were not analyzed in our previous studies, the objective of the present study is to investigate differences in MC infiltration across all three layers of the ascending aortic aneurysm wall between patients with DM and those with AH.

## Materials and methods

### Patients

The study was conducted in accordance with the ethical guidelines established by the Declaration of Helsinki, 1975. Each participant was informed of the study’s purpose and procedures, and written consent was obtained. The study protocol was approved by the National Committee for Medical Ethics (MEC 170/07/13, MEC 110/03/16).

A total of 51 patients indicated for surgical treatment of ascending aortic aneurysm were included in the study. Participants were divided into two groups: those with type 2 diabetes (DM group; *n* ═ 9) and those with hypertension (AH group; *n* ═ 42). Patients with both DM and hypertension, type 1 diabetes, or genetic mutations were excluded from the study.

### Tissue samples

The aneurysmal segment of the aorta was surgically excised and fixed in 10% buffered formalin. Three samples were obtained transversely from the region exhibiting the most significant aneurysmal expansion. These samples were embedded in paraffin and sectioned into a stepped series of 4.5 µm thick slices, with a step height of 50 µm.

The samples were stained with hematoxylin and eosin (HE) and subjected to immunohistochemistry using anti-CD117 (clone: YR145, manufacturer: Cell Marque, catalogue number: 117R-16). The antigen retrieval was performed using CC1 for 56 min, followed by blocking with a pre-primary peroxidase inhibitor. The titer was set at 1/70 with a 24-min incubation, and the detection system employed was OptiView (Ventana, BM ULTRA).

Control samples included liver, appendix, skin, tonsils, prostate, lung, kidney, and melanoma, with fixation times ranging from 1 to 7 days to identify MCs. Additionally, immunohistochemical staining was conducted using anti-von Willebrand factor (vWf) (clone: rb, manufacturer: Dako, catalogue number: A0082). The antigen retrieval utilized protease 1 for 8 min, with no blocking step, a titer of 1/800, and a 32-min incubation. The detection system used was ultraView (Ventana, BM ULTRA), with the same control samples as previously mentioned to identify vascular endothelium, following the manufacturer’s instructions.

### Image analysis

Aortic aneurysm samples were imaged using a light microscope (Nikon Eclipse E400) equipped with a digital camera (Nikon Digital Sight DS-M5) and analyzed using NIS-Elements software (version 3-D). Two independent investigators (AP and MZ), blinded to the experimental groups, manually analyzed the histological slides. Their measurements demonstrated high consistency, with inter-observer differences of less than 10%. In cases of discrepancy, a third pathologist’s evaluation was utilized to reach a consensus. We measured the surface areas of the tunica intima, tunica media, and tunica adventitia [15, 16], and counted CD117-positive cells. The number of MCs was expressed as N/mm^2^. CD117-positive cells were categorized into non-activated intact MCs (characterized by cytoplasm filled with granules and no granules in the surrounding area) and activated degranulated MCs (characterized by fewer granules in the cytoplasm and more granules in the surroundings), along with all MCs (both intact and degranulated).

Vascularization assessment of the tunica media was conducted at a 100× objective magnification, where four regions of interest (ROIs) measuring 1.22 × 0.91 mm were randomly selected. At this magnification, the entire thickness of the media was generally covered. Vascularization was assessed based on the number of ROIs containing blood vessels, employing the following scoring system: 0 – no vessels in any of the four ROIs; 1 – vessels present in one ROI; 2 – vessels present in two ROIs; 3 – vessels present in three ROIs; 4 – vessels present in all four ROIs.

For each aneurysm, we calculated the average values of the measured parameters based on three samples taken from the area of maximum expansion. The average values of all MCs, intact and degranulated MCs, as well as vascularization categories in the DM and AH groups, were calculated and expressed as mean ± SD.

### Statistical analysis

Analyses were conducted using Microsoft Excel 2010 and the Statistical Package for the Social Sciences (SPSS) version 20. The average number of CD117-positive cells per mm^2^ in the tunica intima, media, and adventitia for the DM and AH groups was calculated and expressed as the mean ± SD [15, 16]. Levene’s test indicated that the variances were unequal. The Shapiro–Wilk test revealed that the data were not normally distributed, and the sample sizes differed (AH group: *n* ═ 42; DM group: *n* ═ 9). To identify significant differences between the groups, statistical analysis was performed using the Mann-Whitney *U* test (*P* < 0.05). Additionally, the relationships between the numbers of intact, degranulated, and total MCs and the degree of vascularization in the media were assessed using Spearman’s rank-order correlation (*P* < 0.05).

## Results

### Patients

Patients with aortic aneurysm were categorized into two groups: the DM group (*n* ═ 9) and the AH group (*n* ═ 42). Both groups were predominantly male and primarily comprised non-smokers or former smokers. No significant differences were found between the groups regarding age or plasma levels of triglycerides, total cholesterol, HDL, LDL, creatinine, urea, aortic diameter, and type of aortic valve (either trileaflet or bileaflet). However, systolic blood pressure was significantly higher in the AH group, whereas diastolic blood pressure was elevated in the DM group. Additionally, plasma glucose levels were greater in the DM group (see Table 1).

**Table 1:** Characteristics for all patients in the AH and DM group (average ± SD)

**Variable**	**DM group**	**AH group**	*P* **value Mann–Whitney test**	***R***
	***n* ═ 9**	***n* ═ 42**		
Age (year)	62.56 ± 11.92	57.59 ± 8.33	0.42	0.114
Sex	7 male, two female	35 male, 7 female	0.45	0.106
Smoking	yes 1; no 8 (never smoked 5 stopped smoking 3)	yes 7; no 35 (never smoked 26, stopped smoking 9)		
Total cholesterol (mmol/L)	5.31 ± 1.06	5.01 ± 0.55	0.78	0.040
LDL (mmol/L)	3.72 ± 1.24	3.27 ± 0.68	0.52	0.091
HDL (mmol/L)	1.30 ± 0.45	1.41 ± 0.53	0.79	0.036
Triglyceride (mmol/L)	2.29 ± 1.04	1.71 ± 0.77	0.14	0.208
Creatinine (µmol/L)	82.86 ± 11.85	82.73 ± 11.75	0.94	0.011
Urea (mmol/L)	7.07 ± 1.62	6.77 ± 1.50	0.61	0.071
Aortic diameter (cm)	5.94 ± 0.73	5.54 ± 0.55	0.141	0.206
Aortic valve	tricuspid = 9 bicuspid = 0	tricuspid = 29 bicuspid = 13	0.053	0.271
Systolic blood pressure (mmHg)*	127.22 ± 6.67	138.34 ± 14.21	0.030	0.302
Diastolic blood pressure (mmHg)*	79.73 ± 12.61	73.89 ± 8.94	0.050	0.274
Glucose (mmol/L)*	8.88 ± 0.06	5.51 ± 0.28	0.000	0.656

*Significantly different amount of the component within the DM and AH group; Mann–Whitney test (*P* < 0.05). DM: Diabetes mellitus; AH: Arterial hypertension; LDL: Low-density lipoprotein; HDL: High-density lipoprotein; R: Range; SD: Standard deviation.

### Analysis of intact, degranulated and all MCs in the intima, media, and adventitia between AH and DM groups

In this study, three samples of the aorta from the most expanded segment of the aneurysm were examined for MCs present in the intima, media, and adventitia. Among the patients, seven from the AH group and five from the DM group exhibited no CD117-positive cells in the intima. Within the media, 13 patients from the AH group lacked MCs, whereas only one patient from the DM group exhibited a similar absence. Furthermore, only five patients from the AH group were devoid of MCs in the adventitia, while MCs were present in all specimens from the DM group. It was observed that MCs were more abundant in the outer media and in the connective tissue adjacent to the blood vessels.

Table 2 presents the average counts of intact, degranulated, and total MCs per mm^2^ in the intima, media, and adventitia for both the AH and DM groups.

**Table 2:** Density of MCs (N/mm^2^± SD) in intima, media and adventitia in AH and DM group

**AH (*n* **═** 42)**	**Intima**	**Media**	**Adventitia**
Intact	8.583/ mm^2^± 11.940	0.244/mm^2^ ± 0.359	6.497/mm^2^ ± 7.875
Degran.	3.015/mm^2^ ± 3.970	0.116/mm^2^ ± 0.196	2.454/mm^2^ ± 3.866
All	11.598/mm^2^ ± 15.160	0,359/mm^2^ ± 0.527	8.951/mm^2^ ± 11.539
**DM (*n* **═** 9)**	**Intima**	**Media**	**Adventitia**
Intact	0.659/mm^2^ ± 1.290	0.039/mm^2^ ± 0.036	13.638/mm^2^ ± 5.136
Degran.	0.520/mm^2^ ± 1.286	0.022/mm^2^ ± 0.033	5.327/mm^2^ ± 3.524
All	1.179/mm^2^ ± 2.556	0.061/mm^2^ ± 0.060	18.965/mm^2^ ± 7.688

MCs: Mast cells; DM: Diabetes mellitus; AH: Arterial hypertension; SD: Standard deviation; N: Number; Intact: Intact mast cells; Degran: Degranulated mast cells.

**Figure 1.**
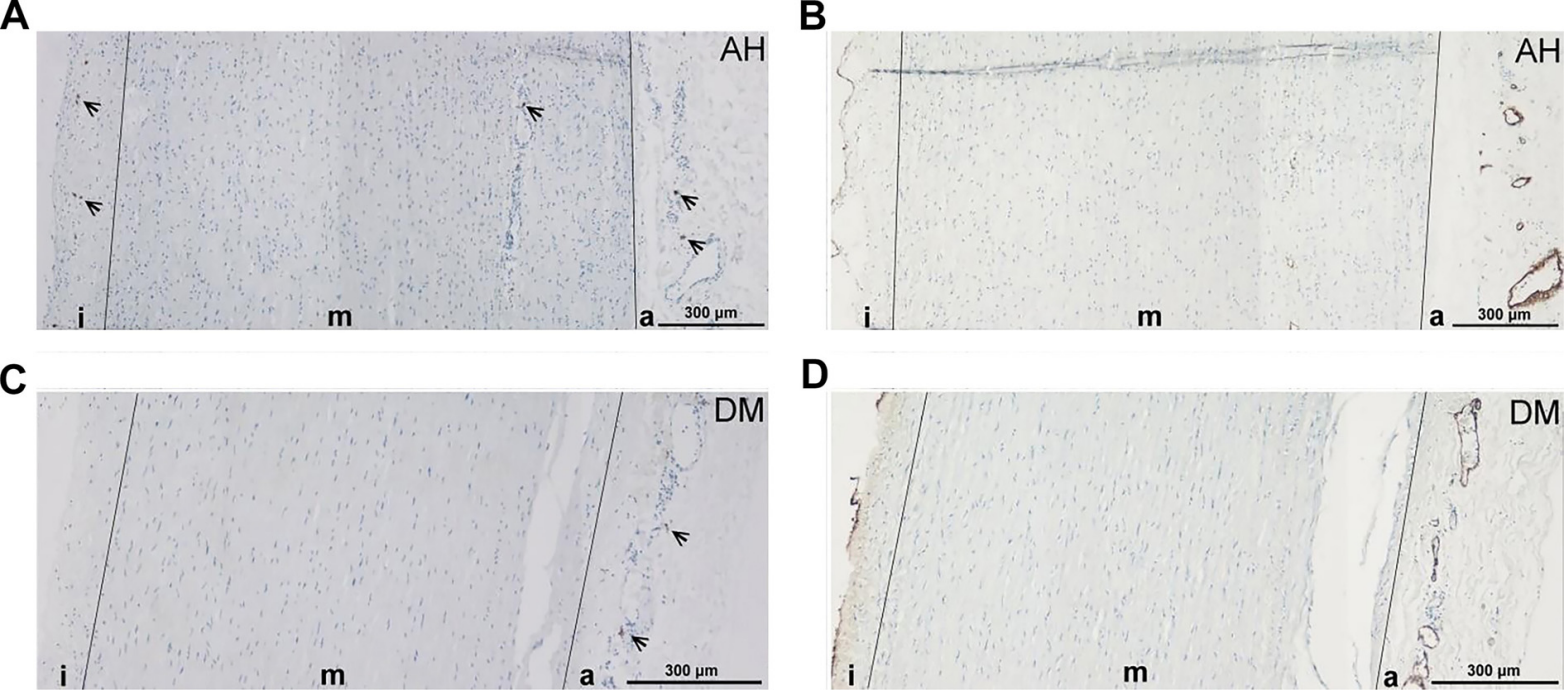
**Representative panoramic immunostained sections of the aneurysmal aortic wall demonstrating mast-cell distribution and medial vascularity in patients with arterial hypertension (AH) and diabetes mellitus (DM).** (A and B) CD117-positive mast cells (arrows); (C and D) von Willebrand factor (vWF)-positive microvessels; i ═ intima; m ═ media; a ═ adventitia. Each panel is a stitched composite encompassing all three layers. Scale bar ═ 300 µm.

A comparison of the AH and DM groups revealed a significantly higher number of intact, degranulated, and total MCs in the intima of the AH group, alongside a significantly lower count in the adventitia (refer to Figure 1 and Table 3).

**Table 3:** The number of MCs/mm^2^ in the intima, media, and adventitia of the ascending aortic aneurysm aortic wall in patients with AH and DM

	**DM (*n* ═ 9)**	**AH (*n* ═ 42)**				
**Variable**	**Mean rank (MCs/mm^2^)**	**Mean rank (MCs/mm^2^)**	*P*	**U**	**Z**	**r**
*Intima*						
Intact*	16.56	28.02	0.032	104.000	−2.140	0.300
Degran.*	17.67	27.79	0.050	114.000	−1.932	0.271
All*	16.44	28.05	0.030	103.000	−2.165	0.303
*Media*						
Intact	22.83	26.68	0.471	160.500	−0.720	0.100
Degran.	21.50	26.96	0.290	148.500	−1.057	0.150
All	24.50	26.32	0.735	175.500	−0.339	0.050
*Advnentitia*						
Intact*	39.56	23.10	0.003	67.000	−3.016	0.422
Degran.*	38.67	23.29	0.005	75.000	−2.819	0.395
All*	40.00	23.00	0.002	63.000	−3.115	0.436

*Significantly different, Mann–Whitney test (*P*< 0.05). AH: Arterial hypertension; DM: Diabetes mellitus; MCs: Mast cells.

Within both the DM and AH groups, the number of intact MCs was significantly greater than that of degranulated MCs only in the adventitia, with no significant differences observed in the intima and media (see Figure 1 and Table 4).

**Table 4:** The density of MCs (N/mm^2^) in the intima, media, and adventitia of the ascending aortic aneurysm aortic wall within patients with AH and DM

	**Intact**	**Degran.**				
**Variable**	**Mean rank (MCs/mm^2^)**	**Mean rank (MCs/mm^2^)**	*P*	**U**	**Z**	**r**
*AH (n = 42)*						
Intima	47.26	37.74	0.068	682.000	−1.823	0.200
Media	45.89	39.11	0.187	739.500	−1.319	0.144
Adventitia*	49.35	35.65	0.010	594.500	−2.575	0.281
*DM (n = 9)*						
Intima	10.11	8.89	0.580	35.000	−0.553	0.130
Media	10.56	8.44	0.380	31.000	−0.878	0.207
Adventitia*	13.33	5.67	0.002	6.000	−3.046	0.718

*Significantly different, Mann–Whitney test (*P* < 0.05). AH: Arterial hypertension; DM: Diabetes mellitus; MCs: Mast cells

### Assessment of vascularity in the media

The vessels were predominantly located in the outer region of the tunica media in both studied groups—AH and DM (Figure 1). No significant difference in vascularity was observed between the DM group (0.852 ± 0.747) and the AH group (1.603 ± 1.204), as determined by the Mann-Whitney test (*P* ═ 0.073) (Table 5).

**Table 5:** Vascularity score in tunica media in AH and DM group (Mann–Whitney test)

	**DM (*n* ═ 9)**	**AH (*n* ═ 42)**				
**Variable**	**Mean rank (MCs/mm^2^)**	**Mean rank** **(MCs/mm^2^)**	***P***	**U**	**Z**	**r**
Vascularity in media	18	27.71	0.073	117.000	-1.791	0.251

AH: Arterial hypertension; DM: Diabetes mellitus; MCs: Mast cells.

### Analysis of vascularity and MCs in media

**Figure 2.**
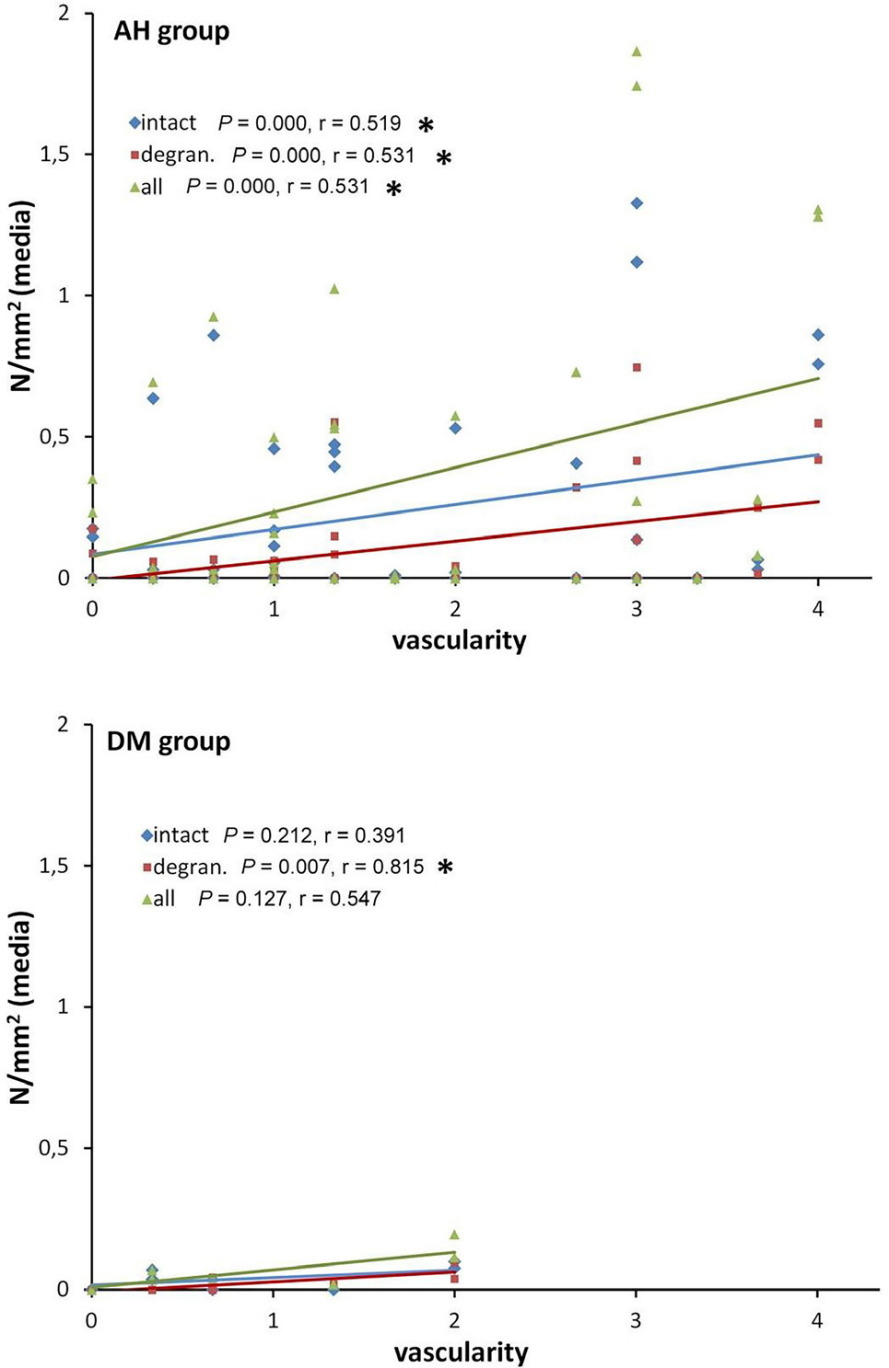
**Spearman correlation between vascularity assessment and number of intact, degranulated and all MCs (N/mm^2^) in tunica media of the AH and DM groups.** An asterisk marks significant correlations (*P* < 0.05). AH: Arterial hypertension; DM: Diabetes mellitus.

In the DM group, a significant positive correlation was observed between vascularity and degranulated MCs (*r* ═ 0.815, *P* ═ 0.007). Conversely, in the AH group, significant positive correlations were found between vascularity and intact MCs (*r* ═ 0.519, *P* ═ 0.000), degranulated MCs (*r* ═ 0.531, *P* ═ 0.000), and the total number of MCs (*r* ═ 0.531, *P* ═ 0.000) (Figure 2).

## Discussion

MCs play a crucial role in host defense against pathogens and are widely distributed throughout the loose connective tissue of the body. They are predominantly located at interfaces between the external environment and internal tissues, particularly in the gastrointestinal tract, respiratory tract, and skin, where they target and destroy antigens such as bacteria, parasites, and viruses [2, 21–23].

Progenitor cells for MCs originate from hematopoietic stem cells in the bone marrow. These cells enter the bloodstream and migrate to various tissues, where they differentiate into mature MCs under the influence of specific cytokines and stem cell factors [24]. The cytoplasm of MCs contains granules rich in pro-inflammatory mediators, including proteases (such as chymase, tryptase, and carboxypeptidase), biogenic amines (including histamine, serotonin, and dopamine), lysosomal enzymes (such as beta-hexosaminidase), glycosaminoglycans and proteoglycans (including heparin, heparan sulfate, chondroitin sulfate, and serglycin), as well as cytokines and growth factors [8, 21, 25, 26]. Upon activation, these granules are released into the environment to combat the antigen.

The most well-known mechanism of MC activation involves the binding of antigens to IgE antibodies attached to FcɛRI receptors on the cell surface [8, 25–27]. This interaction triggers the release (degranulation) of preformed granules, which is characteristic of a type I allergic reaction. In addition to IgE-mediated activation, MCs can also be activated via Fc receptors for immunoglobulin A (IgA) and immunoglobulin G (IgG), complement receptors (e.g., C3a), adenosine receptors, cytokines, chemokines, and various pathogen-associated molecular patterns (PAMPs) [2, 5, 28].

Activated MCs not only release preformed mediators but also synthesize and secrete pro-inflammatory and anti-inflammatory mediators, contingent upon the stimulus and the specific tissue context [2, 8]. In humans, seven transcriptomically distinct MC subtypes have been identified across various tissues, including the trachea, lungs, tongue, lymph nodes, small and large intestines, pancreas, skeletal muscle, skin, bladder, mammary glands, and vasculature [29, 30]. It is hypothesized that the local tissue microenvironment influences MC polarization toward either protective or pathogenic phenotypes. MCs can exert both beneficial and detrimental effects in cardiovascular diseases, such as hypertension, atherosclerosis, myocardial infarction, dilated cardiomyopathy, myocarditis, and thrombosis. One proposed beneficial role of MCs in the cardiovascular system is their promotion of angiogenesis, which is mediated by the release of VEGF-A, tryptase, and the cysteinyl leukotrienes LTC_4_ and LTD_4_ [29].

DM is a chronic systemic inflammatory disease closely linked to atherosclerosis. The inflammatory state associated with DM contributes to endothelial dysfunction, increased vascular permeability, the formation of multiple vascular thrombi, and hemodynamic disturbances [31]. MCs further exacerbate DM-related complications, including atherosclerosis [32], kidney disease [33–36], and cardiomyopathy [37, 38]. Experimental studies indicate that inhibiting MC degranulation or inducing MC deficiency in diabetic mice can prevent the progression of cardiomyopathy [39].

In damaged skin, activated MCs release a variety of mediators, including tumor necrosis factor alpha (TNFα), histamine, vascular endothelial growth factor (VEGF), interleukin 6 (IL-6) and interleukin 8 (IL-8), platelet-derived growth factor (PDGF), transforming growth factor beta (TGFβ), and nerve growth factor. These mediators play a crucial role in promoting wound healing. Activated MCs contribute to the stabilization of blood clots, enhance neoangiogenesis, fibrinogenesis, and epithelialization, increase vasodilation and endothelial permeability, facilitate the recruitment of neutrophils and monocytes to the injury site, and support the activation of keratinocytes and fibroblasts [40, 41].

Diabetic wounds are characterized by delayed closure, impaired angiogenesis, and prolonged inflammation. Experimental models demonstrate that intact skin from diabetic subjects contains a higher proportion of degranulated MCs compared to non-diabetic controls.

Moreover, while MC degranulation increases in response to injury in non-diabetic individuals, this response is absent in diabetics. Notably, pharmacological inhibition of MC degranulation in diabetic models resulted in normalized wound healing. These findings suggest that in diabetes, intact MCs support proper wound repair, whereas MCs degranulation impairs the healing process. This indicates the presence of a functionally detrimental MCs phenotype in the diabetic state [41, 42].

MCs are known to inhabit the intima of healthy aortas and early atherosclerotic lesions, such as fatty streaks; however, their prevalence significantly declines in advanced atheromas and further decreases in AAAs [9, 11, 43]. In contrast, the density of MCs in the media and adventitia increases with the progression of atherosclerosis, reaching its peak in the media and adventitia of AAAs [9, 11, 12].

Notably, a significantly higher number of degranulated MCs have been identified in the media and adventitia of AAAs compared to control and atherosclerotic aortas, where degranulated MCs are nearly absent. In the referenced study, 95% of patients had AH, while only 4% had DM [12].

In the current study, we compared the densities of intact and degranulated MCs in the intima, media, and adventitia of ascending aortic aneurysms among patients with DM and those with AH. AH is well recognized as a prevalent risk factor for aneurysm formation [14], while aneurysms are infrequent in patients with DM who do not also have AH [9, 15–18, 20].

Consequently, the DM group comprised only nine patients. In addition to the limited sample size—particularly within the DM group—our study faced several other limitations, including incomplete patient data such as glycated haemoglobin (HbA1c) levels, duration of diabetes, medication use, and smoking history, the reliance on semi-quantitative vascular scoring, and data derived from a single center.

Our findings indicated that, in comparison to the AH group, the DM group exhibited significantly fewer intact and degranulated MCs in the intima, while displaying a significantly greater number in the adventitia. Within both groups, a notably higher quantity of intact MCs was observed relative to degranulated MCs exclusively in the adventitia, with no such difference detected in the intima or media.

Drawing on data from previous studies [9, 12, 41–43], it can be inferred that the ascending aortic aneurysms in the AH group are less impacted than those in the DM group based on our findings.

It is well established that true aneurysms affect all three layers of the vessel wall, whereas false aneurysms compromise only the media and adventitia.

The thickest layer of the aorta is the tunica media, which, along with the adventitia, provides structural integrity and resistance to the vessel wall. The presence of elastic lamellae within the media allows the aorta to expand during systole as it fills with blood and to recoil during diastole, thereby stabilizing and smoothing arterial pressure in conjunction with left ventricular function. Collagen fibers in the adventitia help prevent excessive stretching of the aorta during systole [44]. The primary mechanical property of the aortic wall is its elasticity, predominantly attributed to the tunica media.

However, our previous studies revealed no significant differences in the composition of the aneurysmal wall—such as the volume density of elastic and collagen fibers, vascular smooth muscle cells, and ground substance—between the DM and AH groups [15, 16]. These findings align with our results, which indicated no statistically significant difference in aortic diameter between the AH and DM groups. It is well established that MCs density correlates with the degree of vascularity in the tunica media of the abdominal aorta under both atherosclerotic and aneurysmal conditions [2, 9, 11, 12].

In our current study, we observed a marginally greater vascularity in the tunica media of the AH group compared to the DM group. This trend of reduced vascularity in the DM group is consistent with previous research indicating that diabetes inhibits angiogenesis and impairs wound healing. In the AH group, vascularity was positively correlated with the total number of MCs, including both intact and degranulated forms. In contrast, in the DM group, a significant positive correlation was found only between vascularity and degranulated MCs.

Activated MCs secrete a diverse range of mediators, including both constitutive and inducible pro-angiogenic factors, such as VEGF [3, 45]. However, research indicates that diabetes suppresses VEGF secretion. Experimental studies reveal that a reduced number of MCs results in lower VEGF levels, leading to delayed wound healing in both diabetic and non-diabetic models. Furthermore, VEGF expression and angiogenesis were significantly diminished in diabetic animals compared to their non-diabetic counterparts during the healing process. Pharmacological inhibition of MC degranulation in diabetic models restored VEGF levels and angiogenesis to those observed in non-diabetic subjects [41, 42].

Consequently, we hypothesized a positive correlation between vascularity and intact (as opposed to degranulated) MCs in the diabetic group.

Despite the seemingly contradictory findings, we hypothesize that MCs may play a beneficial role in the wall of aortic aneurysms. MCs are present in the intima of both healthy and early atherosclerotic aortas, yet they are rare in the adventitia. As atherosclerosis progresses, the density of MCs decreases in the intima while increasing in the adventitia. In cases of advanced atherosclerosis and AAAs, MCs are scarce in the intima but abundant in the adventitia [9, 12]. Furthermore, MCs are crucial for normal wound healing in both healthy individuals and those with diabetes, as their deficiency has been linked to delayed repair [41, 42].

It is essential to recognize that MCs constitute a highly pleomorphic population of inflammatory cells whose behavior is shaped by the local tissue environment. Aneurysm formation is a chronic process, as is the progression of diabetes, and the microenvironments of the intima, media, and adventitia in ascending aortic aneurysms differ significantly from those in experimentally induced skin wounds. Therefore, further studies are warranted to clarify the role of MCs in the pathogenesis of ascending aortic aneurysms in diabetic patients.

## Conclusion

Our findings show that, in diabetic patients, MCs are less abundant in the intima and more abundant in the adventitia compared to patients with AH. This altered distribution pattern suggests that diabetic patients may have a distinct pathogenesis of aneurysm progression compared to those with AH.

## Data Availability

Data supporting the findings of this study are available from the corresponding author upon reasonable request.
